# Current Challenges in Commercially Producing Biofuels from Lignocellulosic Biomass

**DOI:** 10.1155/2014/463074

**Published:** 2014-05-04

**Authors:** Venkatesh Balan

**Affiliations:** ^1^DOE Great Lakes Bioenergy Research Center, Michigan State University, East Lansing, MI 48824, USA; ^2^Biomass Conversion Research Laboratory (BCRL), Department of Chemical Engineering and Materials Science, Michigan State University, MBI Building, 3900 Collins Road, Lansing, MI 48910, USA

## Abstract

Biofuels that are produced from biobased materials are a good alternative to petroleum based fuels. They offer several benefits to society and the environment. Producing second generation biofuels is even more challenging than producing first generation biofuels due the complexity of the biomass and issues related to producing, harvesting, and transporting less dense biomass to centralized biorefineries. In addition to this logistic challenge, other challenges with respect to processing steps in converting biomass to liquid transportation fuel like pretreatment, hydrolysis, microbial fermentation, and fuel separation still exist and are discussed in this review. The possible coproducts that could be produced in the biorefinery and their importance to reduce the processing cost of biofuel are discussed. About $1 billion was spent in the year 2012 by the government agencies in US to meet the mandate to replace 30% existing liquid transportation fuels by 2022 which is 36 billion gallons/year. Other countries in the world have set their own targets to replace petroleum fuel by biofuels. Because of the challenges listed in this review and lack of government policies to create the demand for biofuels, it may take more time for the lignocellulosic biofuels to hit the market place than previously projected.

## 1. Introduction to Biorefineries

**Figure 1:**
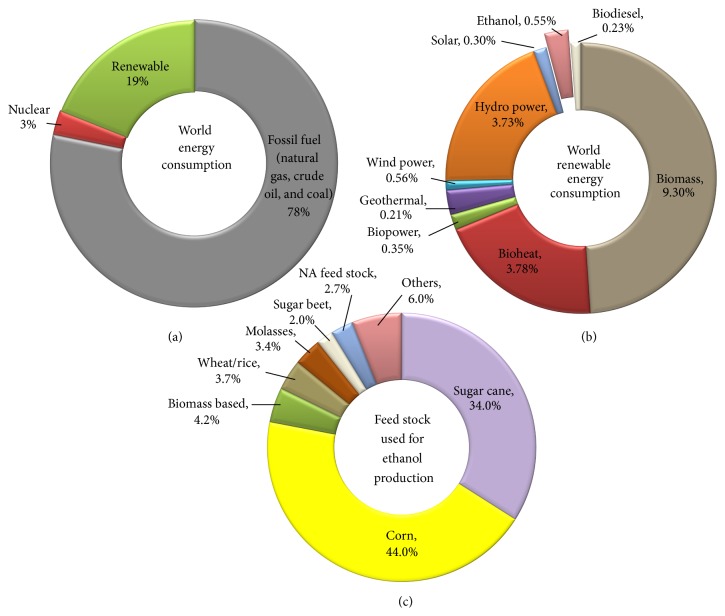
Contribution of renewable energy and biofuels in the total energy consumed in the world in 2011 [4]. Here, (a) gives details about world energy consumption; (b) gives details about world renewable energy consumption, and (c) gives details about different feedstock currently used for ethanol production.

Biorefineries are manufacturing facilities that convert biobased materials (such as agricultural residues) to various products such as food, feed, fuels, chemicals, and energy [1, 2]. Biomass processing is analogous to petroleum refineries, which refine crude oil into several products including fuels (e.g., petrol, diesel, and kerosene) and chemical precursors like butanol for manufacturing different materials [3]. According to renewable energy policy network [4], in 2011, roughly 78% of energy consumed in the world was from fossil fuel, 3% from nuclear energy, and the remaining 19% from renewable energy that is obtained by renewable resources (wind, solar, geothermal, hydrothermal, and biomass). About 13% of the renewable energy is harnessed from carbon rich biobased materials available on earth either by directly burning biomass or by the thermochemical conversion of biomass to heat and power. Currently, the majority of biofuels are produced using sugars extracted from agricultural feedstock or by converting starch into sugars primarily from edible grains (Figure 1). The sugars from both the sources are then fermented into ethanol using yeast [5].

Currently about 85 million barrels of crude oil are processed and used to meet the energy needs of the world. The demands for crude oil are projected to increase to 116 million barrels by 2030 [6]. Since this may result in depletion of crude oil reserves in the world, it is imperative to consider energy sources alternative to crude oil [7]. Biofuels and biochemicals produced using nonedible feedstock such as lignocellulosic biomass provide several benefits to the society [8] like (i) being renewable and sustainable, (ii) indirectly helping carbon dioxide (a greenhouse gas (GHG) that is responsible for global warming) fixation in the atmosphere, (iii) facilitating local economy development and stimulation, (iv) reducing air pollution from burning of biomass in fields and biomass rotting in fields, (v) bringing energy security for countries dependent on imported oil, and (vi) creating high technology jobs for engineers, fermentation specialists, process engineers, and scientists. In the fifties when petroleum processing was invented, the cost of feedstock was cheaper, but the processing costs were higher. Due to technology maturation and advancement, the processing costs have come down; however, depletion of crude oil supply is causing its cost to increase. Currently, it is estimated that conversion of lignocellulosic biomass to biofuels is costlier than its crude oil counterparts. However, it is anticipated that as the technology matures the cost will come down.

**Figure 2:**
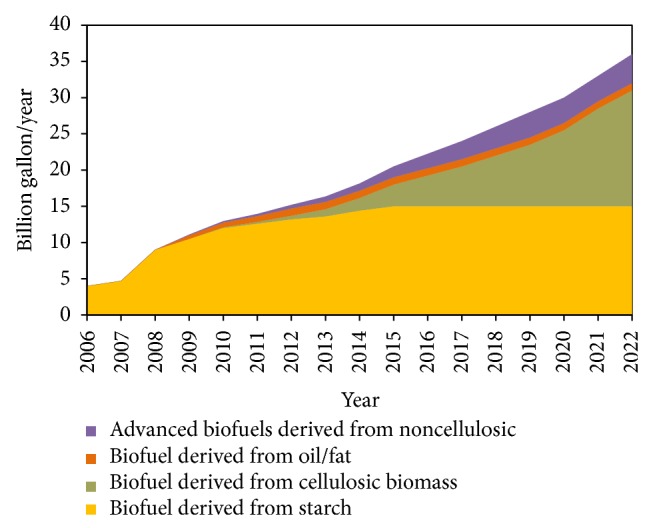
Projected biofuels (gallons/year) production capacity in US. Figure source: Congressional Research Service report number R40155. “Renewable Fuel Standard (RFS) overview and issues” January 23, 2012. The starch based ethanol will saturate at 15 billion gallons/year after 2011 and the amount of cellulosic biofuels production in the US will rise as high as 16 billion gallon/year.

Raising fuel prices hamper the growth of the society as most humans depend on liquid fuels for transportation. Currently, fossil fuels are the only reliable source of energy to run ships, aircraft, trucks, and automobiles, which are essential modes of transportation for the smooth functioning of the society. The US Department of Energy (DOE) and the US Department of Agriculture (USDA) have mandated that 5% of heat and power energy, 20% of liquid transportation fuel, and 25% of chemicals and materials should come from biomass by 2022 [9]. About 36 billion gallons of liquid transportation fuels are needed to meet this ambitious goal. About 15 billion gallons of this biofuel will come from starch based ethanol, while the remaining 21 billion gallons (mostly ethanol) will be produced using cellulosic biomass (Figure 2) [9]. Similar goals were also set by the European Union (EU). Minor amounts of advanced biofuels are projected to come from noncellulosic biofuels by the conversion of gases like carbon monoxide from cement and steel industry using microbial transformation and from transesterification of used cooking oil or animal fat [10]. Other biofuels that are biochemically produced using sugars that could hit the market place are butanol, biofene, and bisabolene [11]. In order to meet these ambitious goals, biorefineries will be successful only if they can fully utilize biomass feedstock's and transform all the components into valuable products [12, 13]. To be highly profitable, biorefineries will need to manage their own energy needs, produce low volume but high value products such as human food, chemical precursors, or medicinal compounds, and produce high volume low value products such as fuels and animal feed.

### 1.1. First Generation Biorefineries

**Figure 3:**
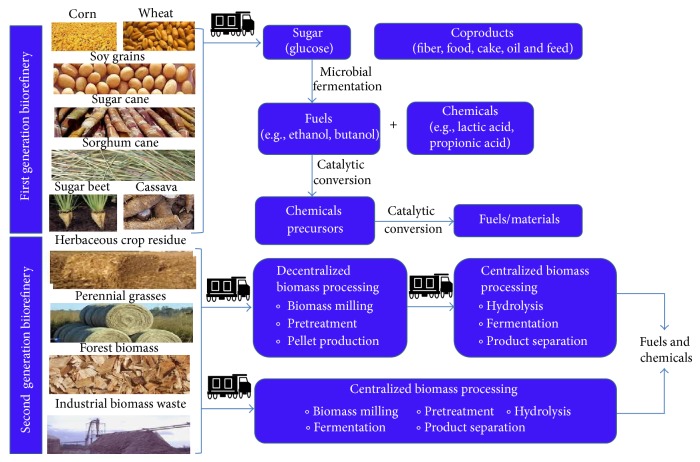
Different feedstocks used in the first and second generation biorefinery for producing biofuels, biochemicals, food, and feed.

The first generation biorefineries used corn, wheat, cassava, barley, rye, soybean, sugarcane, sugar beet, or sweet sorghum as feedstocks (Figure 3). In the cases of sugarcane, sugar beet and sweet sorghum, the sugars are produced by squeezing the stem or by extraction using water [14]. These sugars are subsequently processed using either biological or catalytic transformation to chemicals [lactic acid, propionic acid, poly (3-hydroxyalkanoate acid)/poly (3-hydroxybutyric acid) (PHA/PHB), 1, 3-propane diol (PDO), poly-*γ*-glutamate] or fuels (ethanol, butanol) [15]. On the other hand, processing wheat, rye, corn, or soybean requires additional processing steps (jet cooking using steam followed by starch hydrolysis using enzymes like amylase), after which fuels and chemicals (mentioned above) are produced. Of these, ethanol is the leading biofuel produced around the world and is growing at a steady rate of 20% annually in the US alone [16]. Besides the leading ethanol producers (US followed by Brazil), other countries like France, China, and Canada have shown increasing ethanol productivity using feedstocks such as wheat, cassava, and sorghum juice. In addition to ethanol, several coproducts are produced including food (protein rich fraction, oil, corn steep liquor, and high fructose corn syrup), or animal feed (processed cake, dry distillers grains and solubles (DDGS), and gluten meal) based on the type of processing used (wet or dry milling) [17, 18]. Some other biobased products produced in the first generation biorefinery include adhesives, detergents, dyes, paper, cardboard, polymers, sorbents, paint pigments, and cleaning compounds [19, 20].

### 1.2. Second Generation Biorefineries

Producing fuels from food grade materials has become a controversial topic as there are several million people in the world without sufficient food. Hence, researchers are now focused on developing second generation technologies to produce fuels and chemicals from nonedible feedstocks such as agricultural residue, forest residue, municipal solid waste, industrial waste, and dedicated energy crops [21]. There are two possible routes for converting biomass to biofuels. The first route is “thermochemical” and commonly referred to as biomass-to-liquid (BTL) conversion process. In BTL, the biomass is subjected to pyrolysis or gasification to produce syngas or synthesis gas (mixture of carbon monoxide and hydrogen). These gases are reformed to fuels using either a catalytic process such as the Fischer-Tropsch reactions or by a biological conversion. The second route is a “biochemical” route that transforms sugar polymers present in biomass (cellulose and hemicellulose) to monomeric sugars, which are fermented using microorganisms to produce fuels and chemicals [22] (Figure 3). The third alternative route is a “hybrid” route where a chemical intermediate is produced by a biochemical process and then transformed into higher value products using a thermochemical route. With investments from government agencies, research and development (R&D) has intensified the invention of novel technologies that has accelerated the production of second generation biofuels. Some of the noticeable investments were made by the US and European Union (EU) by establishing several bioenergy centers dedicated to performing basic science for the production of cost-effective biofuels that will lead to novel technologies. In combination, several big companies and startup companies in the US and Europe are developing pilot scale demonstration plants with funding from governments and venture capitalists to scale up these technologies [23].

Recent estimates of biofuel production costs show that second generation biofuels are two to three times more expensive than petroleum fuels on an energy equivalent basis [24]. To bring down the production cost, several challenges in converting lignocellulosic biomass to biofuels and chemicals using biochemical platforms [25–27] need to be addressed. These challenges are in the areas of (i) feedstock production, (ii) feedstock logistics, (iii) development of energy efficient technologies (pretreatment, enzyme hydrolysis, and microbial fermentation), (iv) coproducts development, (v) establishment of biofuel and biochemical standards, (vi) biofuel distribution, (vii) societal acceptance, and (viii) environmental impact minimization. All of these challenging areas require expertise in agronomy, biomass logistics, biomass conversion, process engineering, chemistry, conversion technology, genetic engineering, microbial fermentation, economics, and environmental science. Details surrounding the challenges associated with bringing biofuels and biochemicals to market are discussed in this review.

## 2. Bioenergy Feedstock

Bioenergy plants are broadly classified into two categories, (i) gymnosperms (soft woods like pine, spruce, fir, and cedar) and (ii) angiosperms [*monocots*: all perennial grasses (e.g., switchgrass,* Miscanthus*,* Sorghum*, sugarcane, and bamboo) and herbaceous species (e.g., corn, wheat, and rice);* dicots: *flowering plants (alfalfa, soybean tobacco), hardwoods (e.g., poplar, willow, and black locust)] [28, 29]. The cell wall components (cellulose, hemicellulose, lignin, and ash) vary for different species of plants [30, 31]. Most of the dicots and some monocots have cellulose microfibrils cross-linked with xyloglucans with little arabinoxylan linkages. On the other hand, most monocots consist of glucuronoarabinoxylans as the major cross-linked glycans that are hydrogen bonded to cellulose microfibrils. The lignin [aromatic polymer comprising of syringyl (S), guaiacyl (G), and p-hydroxyphenyl (H)] content and its composition significantly vary in different plant species. Gymnosperms have the highest lignin content and comprises of G and H units. Hard wood species mainly have G and S units and minor amounts of H-units. The monocot grasses have similar amounts of G and S units with significantly higher amounts of H-units than the hard or soft wood plant species [32]. These compositional changes in plant cell wall and differences in ultrastructure greatly influence the pretreatment and the resultant pretreated biomass sugar conversion. For example, ammonia fiber expansion (AFEX) pretreatment process is effective on monocot grasses and herbaceous plant biomass while not as effective on dicots such as poplar and black locust [33, 34]. Also, the same type of biomass harvested from the same field in different years will display changes in biomass composition (due to environmental conditions). This variance poses a challenge in adjusting the processing conditions and directly influences the biofuels yield.

### 2.1. Feedstocks for Biorefineries

**Figure 4:**
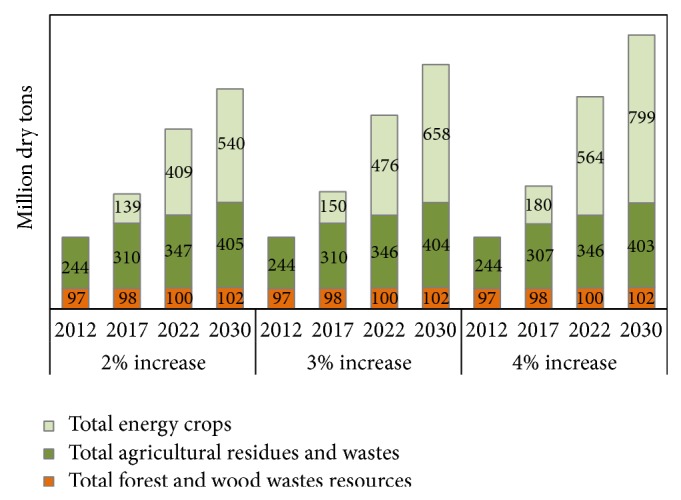
Current and project biomass availability in US based on several assumptions. The data for this plot was taken from billion ton study (2011).

The cost of feedstocks will significantly influence the cost of biofuel production. It is estimated that about 1 billion tons of biomass will be required annually to displace 30% of the US current petroleum consumption. Two reports were prepared by US Department of Energy's Office of the Biomass Program in 2005 and 2011 to evaluate the availability of 1 billion tons of feedstock in the US, [35, 36]. Three major feedstocks were taken into consideration, namely, forest and wood waste resources (such as poplar); agricultural residues (such as corn, sorghum, oat, barley, wheat, soybean, cotton, and rice straws); and energy crops (hybrid sorghum, energy cane,* Miscanthus*, switchgrass and native prairie grass, hybrid poplar, willow, eucalyptus, and pine). Yield assumptions were made based on the crop management, soil condition, and climatic conditions. Three different yield increase assumptions (2%, 3%, and 4%) by year were taken into consideration in these studies (Figure 4).

In 2012, the total amount of feedstock available was 341 million tons. About 70% of this came from agricultural residues and 30% from forest residues. The forest biomass showed a marginal increase in yield, while the agricultural biomass showed a modest increase with time due to projected improvements in agronomy practices. Energy crop plantations, particularly switchgrass [37, 38], poplar [39], Bermuda grass [40], sweet sorghum [41], and* Miscanthus* [42, 43], were tested in several test plots and the biomass will be made available in market by 2017. By 2022, dedicated energy crops will be one of the major feedstocks for bioenergy conversion. A challenge will be to convince farmers who cultivate grains for living to switch to bioenergy feedstocks. Farmers can be assured of buy back guarantees to overcome their reluctance and to remove the risk of failure to sell their cultivated products.

The estimated total biomass that may be available in 2030 based on the three different yield increase assumptions (2%, 3%, and 4%) would be 1047, 1164, and 1304 dry tons, respectively. Other potential high yielding biomass feedstocks available for conversion are agave bagasse (tequila industry in Mexico and Australia), Erianthus (a cane variety grown in south Asian countries like India), Napier grass (widely used in Japan as animal feed), date palm (available in Middle Eastern countries and Africa), and oil palm empty fruit bunch (widely available in Indonesia and Malaysia) [44]. In addition to these feedstocks, several industrial wastes such as rice husk, cotton gin waste, wheat dust, orchard and vineyard pruning, and fruit waste could be used as potential feedstocks for producing biofuels and biochemicals. However, these materials are seasonal and need to be available in large quantities to be considered as feedstocks for biorefineries.

### 2.2. Biomass Harvesting

Biomass harvesting is an energy intensive process that requires large machinery and demands large amounts of fuel for transportation [45]. Different types of machinery are used for harvesting different types of biomass and the cost of harvesting may be influenced by the type of machinery used [46]. Woody biomass is harvested as felled-timber and then chipped or cut in to different lengths, while energy crops are usually harvested in a single pass with three steps, which are cutting, raking, and baling [47]. Agricultural residues are harvested by collecting and baling the biomass residue after harvesting the grains [48]. Most of the moisture conditioning of the agricultural residue is done in the field prior to baling [49]. Soil contamination of biomass is considered as one of the biggest challenges in biomass harvesting. Other key challenges are the moisture content of biomass (influenced by local weather conditions during fall harvest season) and the amount of biomass that can be harvested from the field (dictated by the tilling conditions used and soil requirements to maintain the biomass productivity during the subsequent years) [50].

### 2.3. Biomass Yield

About one-third of biofuel production cost is associated with biomass cost. The cost of biomass ($ per ton) is directly proportional to the yield (ton per ha) [51], which is influenced by soil fertility, location, and genetics. Many plant breeding and genetic engineering techniques have been applied to increase the yield of several potential energy feedstocks including switchgrass, poplar, forage sorghum, and* Miscanthus* [52, 53]. The following are gene alterations by which growth can be promoted in different energy crops: (i) photosynthetic genes, (ii) transcription factors, (iii) cell cycle machinery, (iv) hormone metabolism, (v) lignin modification [54], and (vi) micro-RNA. In addition to these genetic manipulations, biomass yield can be improved by manipulating pathways in both abiotic and biotic stress [55, 56]. Some of the potential feedstocks that will be used in biorefineries and their current yield are shown in Table 1. A combination of transgenesis, classical plant breeding, and modern agricultural practices have been shown to increase the yield of biorefinery feedstocks. These genetically modified crops may need to have their safety ensured to gain public acceptance.

## 3. Biomass Supply Chain and Logistics

The biomass supply chain will include several key processing steps, which are (i) collection, (ii) storage, (iii) preprocessing (densification by compaction, pelleting, and briquetting), (iv) transportation (from field to biorefinery), and (v) postprocessing at the biorefinery [47, 57]. These supply chain steps will directly impact the cost of feedstock delivery. Texture variance, seasonal availability, low bulk density, and distribution over a large area are major challenges in transporting lignocellulosic biomass to biorefineries [58]. The low density of biomass is known to occupy more volume and requires more transportation carrier space and hence is reported to influence the transportation cost. Transportation cost is also influenced by the moisture content, distance from the field to biorefinery, available infrastructure, available on-site technology, and the mode of transportation (rail or road) [59, 60]. It has been documented that the cost of biofuels depends greatly on the biorefinery size, which will require optimization based on the location and feedstock(s) [61–63].

### 3.1. Biomass Densification

Biomass densification is an energy intensive process done after size reduction by traditional milling processes followed by a compaction process to increase the density by severalfold [64]. During the densification process, the biomass is packed through elastic and plastic deformation. Several technologies are currently developed to densify the biomass and among them the screw press is reported to consume the most energy [65]. In order to improve the biomass binding characteristics, milled biomass should be either heated at an elevated temperature (100–130°C) where the lignin melts to act as a natural binder or mixed with external binders like soluble sugars, fat, starch, protein, or lignosulphonate [66]. Lignosulphonates are preferred when biomass pellets are used for thermochemical applications, where they increase the calorific value of the substrate. Since lignin is a recalcitrant molecule that inhibits enzyme and microbes, it should be avoided when the pellets are used for biochemical transformation applications. However, adding external binders is an additional cost for making biomass pellets. Steam conditioning, steam explosion, and AFEX pretreatment also facilitates the lignin that is buried in the biomass to rise to the surface and aids in the densification process. In addition to temperature, milling speed and pressure are key parameters that need to be optimized to get high quality pellets [67]. Besides these another densification method that is widely used in the industry is the agglomeration technique [68]. It is important to note that different feedstocks pelletize differently and may require different size and thickness of pellet dies [69].

### 3.2. Centralized versus Decentralized Biomass Processing

**Figure 5:**
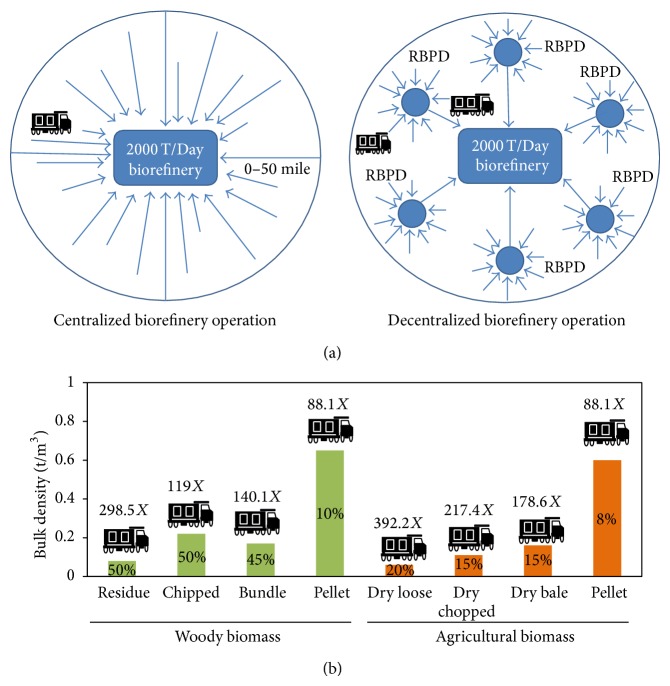
Biomass transportation in a centralized and decentralized biorefinery operation (a). Various forms of biomass that will be used in a 2000 ton per day biorefinery and how many times the truck has to transport this biomass is shown (b). Moisture content of each of this biomass is given inside the bars.

Feedstock location is limited to 50 mile radius for the economic operation of a biorefinery that processes 2000 tons biomass/day (Figure 5(a)). Since biomass has a low bulk density and should not be stored at facilities with high moisture content [70, 71], it is more logical to process the biomass (size reduction, pretreatment, and densification) near the field and store them [72, 73] in a decentralized processing facility. Densified biomass with less than 10% moisture will be stable for several months and requires a smaller foot print for storage. This is also advantageous to the biorefinery since feedstock can be transported when needed, resulting in guaranteed feedstock supply all through the year. The concept of regional biomass processing depots (RBPDs) or in other words cooperative systems owned by farmers that process the biomass was first proposed by researchers at Michigan State University [74]. This cooperative model was extensively studied by the University of Tennessee Biofuel Initiative and benefited Dupont cellulosic ethanol (DCE) and the Idaho National Laboratory (INL) by supplying different and mixed feedstocks [75]. The RBPD model can also be expanded to produce other coproducts like lignin, bio-oil, biochar, torrefied biomass, and proteins and could be integrated for in-house energy production using biogas generation [76]. Setting up RBPDs near the cultivation site will generate jobs and will enable construction of infrastructure in rural areas. Although the agriculture cultivation is seasonal, using integrated processing will support operations throughout the year. Life cycle analysis has shown that processing the biomass in RBPDs (decentralized operation) and transporting it to biorefineries reduces greenhouse gas emissions when compared to transporting it to a centralized biorefinery [77, 78].

There are several challenges in establishing an RBPD, which include (i) identifying energy requirements for the size reduction, pretreatment, and densification operation that will require additional investment to create the infrastructure, (ii) disadvantage of a small scale operation due to economy of scale, and (iii) establishment of the facility near rail lines or creating new rail lines may be required for economic transportation of biomass [74, 75]. For RBPDs a dry-to-dry pretreatment process such as AFEX, disk milling, extrusion is advantageous compared to wet pretreatment processes that may require large amounts of water such as dilute acid, steam explosion, and hot water pretreatment [73].

### 3.3. Biomass Transportation

Woody biomass can be transported in four different forms: whole tree residue, wood chip, bundle, and pellet. Agricultural biomass can be transported as loose, chopped, baled (square or round), or pelleted forms. The density of the biomass can significantly change depending on the level of processing that the feedstock undergoes [79, 80]. When compared to loose biomass, the density of pellets can increase as high as 10 times for agricultural biomass and 8 times for woody biomass (Figure 5(b)). One option available to transport biomass is by trucks. The dimensions of heavy duty trucks can vary from place to place. For a base case scenario, the pay load (maximum load) of a truck is assumed to have a max of 22.7 tons at a volume of 70 m^3^ [81]. This truck could carry 5.1, 9.2, 11.2, and 22.7 tons of agricultural biomass (loose, chopped, baled, and pelleted, resp.), or 6.7, 16.8, 14.2, and 22.7 tons of forest residues (chipped, bundled, and pelleted woody biomass, resp.). Assuming that a biorefienry requires about 2000 tons of biomass/day for making biofuels and biochemical [61], transporting agricultural biomass or woody biomass as pellets would require 88 trips/day versus loose biomass, which would take 392 and 298 trips/day for agricultural biomass and woody biomass, respectively. The cost of biomass transportation is expressed as a fixed cost ($ per ton) and variable cost ($ per ton km) using round trip distance (km). The cost of transportation will be higher as the distance from the biorefinery to biomass storage location increases [57].

### 3.4. Biomass Storage

Harvested biomass can be protected from rain by storing in a building or by covering using a polythene wrap before being shipped to the biorefinery. Biomass feedstocks are vulnerable to microbe aided decomposition facilitated by the moisture present in the biomass after harvesting [82]. When the moist biomasses are stored under aerobic condition in the shed or covered with polythene tarp, several aerobic microbes including fungal infestation could take place. This could possibly reduce the sugar content of the biomass [83]. Storing biomass at <10% moisture (dry weight basis) is recommended for long term stability. In contrast, 20–30% moisture is recommended during biomass pellet making. Hence, biomass should be dried before and after densification (harvested biomass has >30% moisture) [60]. Furthermore, precautionary measures are necessary to prevent self-ignition of biomass during storage [84].

## 4. Biomass Pretreatment

Lignocellulosic biomass is a complex matrix comprised of cellulose (35–50%), hemicellulose (20–35%), lignin (15–20%), and other minor components [ash, protein, minerals, pectin, etc.] (15–20%) [85, 86]. Biomasses from plants are naturally recalcitrant, having evolved to protect themselves against invading microorganisms. In the wild, when the plants die, they are decomposed by microbes under moist conditions. However, since plant biomasses are recalcitrant, the rate of decomposition is slow and may take several months to years to completely degrade dead plants. On the other hand, in a biorefinery, conversion of biomass to biofuels has to be done in days. In order to increase the accessibility of cellulose and hemicellulose, the hemicellulose-lignin complex cross-links must be broken. Many pretreatment processes have been developed to accomplish this step.

Prominent pretreatment methods include (i) physical pretreatment (size reduction by grinding, milling [87], extrusion at elevated temperature [88], etc.); (ii) chemical pretreatment under alkaline conditions (AFEX [89], ammonia percolation process (ARP) [90], soaking aqueous ammonia (SAA) [91], NaOH [92], alkaline hydrogen peroxide [93], lime [94], alkaline wet oxidation [95], steam explosion under alkaline condition, etc.), neutral conditions (ionic liquid [96] liquid hot water [97], ozonolysis [98], super critical water), or acidic conditions (dilute sulfuric acid [99], organic acid [100, 101], concentrated acid [102], organosolv under acidic condition [103], SPORL [104], etc.); (iii) physiochemical pretreatment (steam explosion under acidic conditions [105], super critical CO_2_ [106, 107]; and (iv) biological pretreatment [108, 109]. Excellent reviews have been published in the past five years explaining different pretreatment options that have been developed and have potential for commercialization [110–116]. Rather than going into the details of the processing steps, the conditions used during the pretreatment process and the advantages and disadvantages of each process are given in Table 2.

Not all pretreatments are created equal. Pretreatment processes that show potential commercialization should satisfy most of the criteria below.

- A pretreatment process that opens up the cell wall and brings lignin to the surface has the potential to efficiently densify after pretreatment without adding any external binding agents and therefore is ideal for decentralized biomass processing. This will also help to increase the durability of biomass for long term storage. Some of the pretreatments that can fall under this category include AFEX, wet oxidation, and extrusion at elevated temperature.
- Densified pretreated biomass that has dual application (fertilizer, soil amendments, animal feed, and biomass composites) in addition to using them as biorefinery feedstock can penetrate the market quickly.
- Pretreatment processes that generate lesser amount of degradation products, which are toxic for downstream processing, namely, enzyme hydrolysis and microbial fermentation. Processes that generate large amount of toxic degradation products require large amount of water to remove the toxins from the pretreated biomass making the process more expensive.
- Pretreatment processes that can be scaled up to meet the biorefinery needs of handling more than 2000 tons per day or more. The capital cost of pretreatment reactor could increase rapidly based on the pretreatment conditions and the catalyst used. For example, in microwave radiation [117], gamma irradiation required specialized reactor system and also should undergo several additional safety regulations before this technology could get commercialized. Acid pretreatment requires hastelloy reactors to overcome corrosion, while most other pretreatments require reactors made of stainless steel.
- Pretreatment processes that use less energy because less energy results in less processing cost and vice versa.
- Pretreatment processes that use cheap chemicals. Using an expensive chemical like ionic liquid requires additional recovery step which can dramatically increase the processing cost of pretreatment.
- Preservation of lignin during pretreatment. Portion of lignin will be used to create heat and electrical energy to drive different processing steps in biorefinery. Pretreatments like alkaline hydrogen peroxide and ozonolysis have the tendency to degrade lignin and hence the energy density of lignin will reduce.
- Pretreatments done at moderate temperatures and pressures are highly preferred. This has implications on the cost of the reactor and the safety measures that companies have to take before these technologies can be implemented. Some of the pretreatment processes that use supercritical fluids (water and CO_2_) operate at a very high pressure and require additional cost while building the reactors.
- Using less hazardous chemicals is preferred. For example, chemicals like hydrofluoric acids should undergo additional safety steps to avoid accidents during biomass processing, which will increase the processing cost.
- Catalyst recovery during pretreatment process is very important for the environment. Although this will slightly increase the processing cost, there will be energy savings overall, since less amount of chemicals will be used during the process. Moreover, most of the catalyst (acid or base) is soluble in water and end up in the waste stream. They should be neutralized with a base or an acid for salt formation. Since most of the biorefinery will be reusing the water, removing these salts from the water will eventually increase the processing cost.

Pretreatment can drastically change the properties of the pretreated material (specific surface area, cellulose crystallinity index, degree of polymerization, lignin content in biomass, acetyl content in biomass, etc.). Effective pretreatments increase the rate of enzyme hydrolysis and significantly decrease the amount of enzymes needed to convert the biomass into sugars, which can be utilized by microorganisms. Since lignin is highly recalcitrant and is responsible for unproductive binding of enzymes, the efficiency of conversion of sugar is influenced by the amount of lignin present in pretreated biomass. Removing lignin during the pretreatment process will enable recovery and reuse of enzymes resulting in significant cost savings.

### 4.1. Physical Pretreatment

Physical pretreatments include mechanical processing and extrusion where the objective is to reduce the particle size but increase the surface area. For example, disk milling/grinding results in particle sizes of 0.2–2 mm and chipping results in particle sizes of 10–30 mm [118]. Extrusion processes subject the biomass to mixing and shear forces by extruding the biomass under elevated temperatures. The extrusion processes result in shortening of fiber and defibrillation, which increases the accessibility to enzymes during hydrolysis [119]. Grinding and chipping will be adopted in the biomass processing centers prior to thermochemical pretreatment of biomass. Other milling processes include ball milling, hammer milling, and colloid milling. All of these milling processes are highly energy intensive and are unlikely to be used in a biorefinery context.

### 4.2. Chemical Pretreatment

Chemical pretreatments are carried out at acidic, neutral, or basic conditions. Under acidic conditions (using mineral acids such as H_2_SO_4_, HCl, H_3_PO_4_, and HNO_3_ or organic acids like fumaric, maleic, and acetic acid), hemicellulose is solubilized to monomeric xylose leaving the cellulose and lignin behind. The cellulose can then be enzymatically digested to monomeric sugars [120]. The concentration of acid, the pretreatment temperature, and residence time will influence the formation of degradation products like HMF, furfural, and several phenolic lignins, which are inhibitory for downstream processing (enzyme hydrolysis and microbial fermentation). Organic acids are known to produce less degradation products when compared to mineral acids. Pretreatment at neutral conditions are carried out by using ionic liquid (IL). IL is a promising pretreatment process capable of solubilizing both cellulose and hemicellulose that can then be regenerated using antisolvents like water or organic solvents. The cost of IL and catalyst required for pretreatment are major bottlenecks preventing commercialization of this technology [121, 122]. Organosolv is another pretreatment process that can be carried out either at neutral or acidic conditions using organic solvents like methanol, ethanol, acetone, ethylene glycols, and tetrahydrofurfuryl alcohol. Acidic organosolv processes are preferred and when carried out as two-stage process can efficiently fractionate the biomass into hemicellulose, lignin, and cellulose [123]. Other prominent neutral pretreatments include ozonolysis (using ozone as a catalyst to break down lignin), wet oxidation (using oxygen in the presence of Na_2_CO_3_), microwave assisted pretreatment, and hot water pretreatment (using water at higher liquid to solid ratio resulting in solubilization of hemicellulose as oligomeric sugars).

Alkaline pretreatments effectively swell and increase the internal surface area of cellulose, decrease crystallinity, cleave lignin carbohydrate complexes (L-C-C), and solubilize lignin. During this process, minor amounts of hemicellulose and cellulose [124] are solubilized. Alkaline processes produce lower amounts of sugar degradation products when compared to acidic pretreatment processes. Some of the prominent alkaline pretreatment processes employ catalysts such as NaOH, KOH, ammonia, or lime. The pretreatment is either done at low temperature for a long residence time or at elevated temperature for a short residence time. The former pretreatment processing conditions are more economical, while the later processing conditions are expensive but could handle larger volume of samples in a short period of time. Strong alkali species like NaOH and KOH cleave ester and ether bonds, while weak alkali species like ammonia cleave only ester bonds. In some cases, oxidants like oxygen or hydrogen peroxide are used to improve the performance of the pretreatment by efficiently removing lignin [125]. AFEX pretreatment is a dry-to-dry process depositing most of the cleaved lignin near the surface of the biomass and does not have any liquid stream generation during the process [126].

Two other prominent physiochemical pretreatment processes widely used are steam explosion (in the presence or absence of SO_2_) [127] and CO_2_ explosion (using super critical CO_2_ that produces carbonic acid). Instead of a single step steam explosion (1.2 MPa/8 min), a two-step steam explosion (1.1 Mpa/4 min and intermediate separation of fiber cell—1.2 Mpa/4 min) was found to increase the sugar conversion during enzyme hydrolysis by 13% and also improved fermentation [128]. The SO_2_ catalyzed steam explosion process utilizes both chemical (by hydrolyzing acetyl groups that are present in hemicellulose) and physical (by disrupting fibers during explosive release of pressure) effects. Though this process has been successfully demonstrated on hard wood and herbaceous residues, it is not as effective for soft woods [118]. The CO_2_ explosion is reported to be more cost-effective and produce lesser degradation products when compared to steam explosion [129]. However, both the processes require high pressure reactors that can significantly increase reactor and processing costs.

### 4.3. Biological Pretreatment

Microorganisms like brown, white, and soft-rot fungi are used to pretreat biomass in the biological pretreatment process. Microbes are very effective in degrading lignin with the help of lignin degrading enzymes (peroxidases and laccases) [130]. Biological pretreatments are operated at mild conditions and require low capital costs when compared to expensive reactor systems required for physical/chemical pretreatment processes. However, the biological process is a relatively slow process requiring several days to pretreat the biomass. Furthermore, the sugar conversion after the microbial pretreatment process is lower when compared to chemical pretreatment. In many cases, biological pretreatment followed by chemical pretreatment is found to be effective and requires less severity pretreatment conditions for effectively hydrolyzing the biomass [131].

### 4.4. Catalyst Recovery

Most of the catalysts (either acid or base) used in the pretreatment processes are miscible in water and end up in the waste water stream. Recovering catalysts from the dilute water stream is an energy intensive and expensive process. Either chemical precipitation or an expensive ultrafiltration method is used to recover the catalyst. In some cases, the catalysts used in the process are very low in concentration (e.g., dilute sulfuric acid, dilute ammonium hydroxide, etc.) and in such cases catalyst recovery is not necessary. Neutralizing the waste water generated during pretreatment process with acid or base addition results in salt formation. These salts can cause additional cost when recycling the water for subsequent processing steps. The above considerations are not necessary for pretreatment processes using ammonia [132, 133]. Since ammonia is a volatile alkali, it can be recovered and reused as in the AFEX process. When organic solvents (e.g., ethanol in organosolv pretreatment process) are used they have to be recovered using energy intensive distillation processes. In the case of mechanical processing, microwave processing, wet oxidation, ozonolysis, hot water, supercritical water, or carbon dioxide usage, there is no catalyst involved and hence there is no expensive catalyst recovery step required. However, these processes need expensive reactor systems. Using phosphoric acid pretreatments results in formation of highly digestible amorphous cellulose, however, recovery of phosphoric acid from water is a very expensive process [134].

### 4.5. Influence of Pretreatment on Cellulose Crystallinity and Sugar Conversion

Plant cell wall consists of macromolecule cellulose I_*β*_ which is tightly packed with glucan chains that are held together with inter- and intramolecular hydrogen bonding [135]. The glucan chain length can change anywhere between 100 and 10,000 glucose units, depending on the source of plant biomass [126, 136]. Because of the close packing of the glucan chain (two-chain monoclinic unit cell), the cellulose microfibril is found to be highly recalcitrant. Most of the pretreatments listed in Table 2 result in marginal increase in cellulose crystallinity with some reduction in the degree of polymerization. Some pretreatments result in changes in the crystal structure (different polymorph of cellulose). These changes in the crystal structure happen during pretreatments using chemicals like NaOH, IL [137, 138] (cellulose II), ammines and ammonia (at higher ammonia to biomass ratio greater than 3 : 1) (cellulose III_I_) [135, 136], and glycerol (cellulose IV) [139]. Pretreatment using 85% phosphoric acid results in the formation of amorphous cellulose [134]. The cellulose crystal packing greatly influences the rate of enzyme hydrolysis (cellulose IV > cellulose III > cellulose II > cellulose I). In the case of cellulose IV, the hydrogen bonding is completely distorted and the enzymes are able to depolymerize the cellulose [28]. Cellulose III has distorted crystal packing exposing the hydroxyl group to the surface and making it more hydrophilic than cellulose I, which allows the enzyme to remove efficiently and bind to the cellulose. It is widely believed that when more enzymes bind to the cellulose substrate, the conversion will also be more.

### 4.6. Influence of Pretreatment and Degradation Products on Downstream Processing

To allow enzymes to have easy access to the sugar polymers, pretreatment helps to open up the complex plant cell wall. During the pretreatment process, different chemicals are used as catalysts to cleave chemical linkages (ester and ether) and release small organic molecules. The most prominent small molecules that are produced during hydroxyl ion based ester linkage cleavage are acetic acid, ferulic acid, and coumaric acid. Corresponding amides are produced if ammonium ion is responsible for cleaving the ester linkages. Other prominent aliphatic acids that are produced include formic, lactic, succinic, fumaric, aconitic, levulinic, and itaconic acids. Some of the lignin degraded products include vanilic, caffeic, syringic, and synaptic benzoic acids. Aldehydes released during pretreatment include vanillin, 4-hydroxyacetophenone, 4-hydroxybenzaldehyde, and 3,4-dihydroxylbenzaldehyde [33]. The most prominent sugar degradation products produced during acid hydrolysis include 2-furoic acid, 4-hydroxymethyl furfural, and furfural. It has been estimated that the amount of degradation products produced during acid pretreatments is two times higher than alkaline pretreatments [140]. The concentration of the degradation compounds could significantly change if the severity (higher temperature, higher catalyst concentration, and longer residence time) of the pretreatment changes. These degradation compounds have been found to inhibit enzymes during hydrolysis and microbes during the fermentation processes.

It is impossible to avoid degradation product formation during pretreatment. Hence, researchers are trying to identify enzymes and microbes that are less inhibited by these compounds or to develop methods to remove these compounds by using water or solvent based extractions. In the case of acid pretreatment, expensive detoxification steps are followed prior to microbial fermentation [141, 142]. They include passing the hydrolysate through activated charcoal (which absorbs organic compounds) or raise the pH to 10 and then reduce to 5 to precipitate degradation compounds as in overliming. However, these additional processes add cost and are not practical in scaled up biorefineries. Another promising approach to consider is to remove the degradation products and lignin molecules using the extractive ammonia pretreatment process [143]. In this case, appropriate cosolvents are added along with the ammonia during the pretreatment thereby increasing the solvent to biomass ratio from 3 : 1 to 6 : 1. Depending on the type of solvent and water used in the process, some hemicellulose is lost during the pretreatment. However, up to 45% of lignin can be removed by this approach. Other prominent pretreatment that removes lignin are ionic liquid, organosolv, and ARP processes.

## 5. Enzyme Hydrolysis

Microorganisms secrete enzymes to degrade biomass for producing monomeric sugars for their own survival. There are two ways microbes use the biomass degrading enzymes in a natural environment. The first is cellulosomal enzyme system (a complex mixture of enzymes that are docked to cohesive and doctrine domains which are anchored on the surface of the organisms) [144]. The second is the free enzyme system (where enzymes are secreted as individual components) to act on biomass substrates [145]. The free enzyme system is easy to duplicate and is widely used for biomass conversion in biorefineries. Free biomass degrading enzymes are produced in large scale by commercial companies using fungus or bacteria. The microbes are fed with agricultural residues (grains, hulls, and several biomass waste generated in the industry). This cocktail contains 40–50 different biomass degrading enzymes which can be classified into three major categories: cellulases (which degrade cellulose), hemicellulase (which degrade hemicellulose), and pectinase (which degrade pectin) [146]. Cellulase constitutes about 70–85% of the cocktail and hemicellulose and pectinase constitute the remaining 15–30% depending on the organisms and choice of substrate. Another class of enzyme that could also play an important role in biomass deconstruction is ligninases (which degrades lignin).

Different types of enzymes are needed to cleave different types of bonds during hydrolysis of lignocellulosic biomass due to the complex network of cellulose and hemicellulose [147]. Such enzymes are called molecular scissors and produce specific monomeric sugars from complex carbohydrates. Several excellent articles have been published in the past documenting specific activities of each enzyme and how they work synergistically during hydrolysis [148–151]. For example, cellobiohydrolase I (CBHI) acts on the reducing end of cellulose chains, cellobiohydrolase II (CBHII) act on the nonreducing end of cellulose chains, endoglucanase (EG) act on the amorphous region of cellulose, and beta-glucosidase (*β*G) act on cellobiose to produce monomeric glucose. To be most effective, individual enzymes need to be present in the appropriate ratio due to their synergistic nature.* Trichoderma reseei *(a filamentous fungus) produces high concentrations of enzymes up to 100 g/L and is widely used by commercial companies to produce biomass-degrading enzymes (Novozyme and Genencor) [152]. Other thermophilic fungal and bacterial enzymes have recently been introduced to the market, which perform at higher temperatures to prevent* Lactobacillus *(converts sugars to lactic acid) contamination [153, 154].

### 5.1. Cost of Enzymes

The enzyme quantity required to hydrolyze lignocellulosic biomass is onefold higher than for starch. This is primarily because lignocellulosic biomass is naturally recalcitrant with a complex ultrastructure. Lignin in plant cell wall and degradation products produced during pretreatment are responsible for deactivating enzymes and hence increased amounts of enzymes are required [155]. In the past decade, commercial enzyme companies have made significant progress in producing new generations of enzymes with higher specific activities and lower cost using different biotechnology and process engineering approaches [156]. However, technoeconomic analyses have shown that more progress needs to be made. The cost of enzymes is one of the driving factors for current research [157]. Already several approaches have been adopted to produce highly active enzymes. These include (i) identifying novel multifunctional enzymes that can hydrolyze different types of polysaccharide linkages [158], (ii) identifying novel enzymes that have superior activity [159], (iii) producing novel enzymes using different genetic engineering approaches [160], and (iv) direct evolution of enzymes [161]. Another novel approach is expressing enzymes in plants that could be extracted and used after pretreating the extracted biomass for producing fermentable sugars [162]. Enzymes could also be produced directly in biorefineries rather than producing them in a centralized location. Producing enzymes on-site at biorefineries would eliminate the need for concentration, storage, and shipping and could reduce the production costs by using pretreated substrates already available at the biorefinery [163].

### 5.2. Enzymatic Hydrolysis Time

The time taken to completely hydrolyse biomass to monomeric sugars depends on several factors: lignin content in biomass [164], pretreatment effectiveness [136, 165], cellulose crystallinity [166], substrate concentration, and enzyme activity. Removing hemicellulose during pretreatment (dilute acid, acid catalyzed steam explosion, organosolv, and phosphoric acid pretreatment) significantly reduces the recalcitrance of biomass allowing for a fast rate of sugar conversion [166–168]. When looking at the rate of enzymatic hydrolysis on AFEX pretreated corn stover (where there is no hemicellulose removal), biphasic kinetics are present with a fast hydrolysis phase that produces 70% sugar conversion in the first 24 hours and then a slow hydrolysis phase requiring more than 6 days to completely depolymerize the remaining complex carbohydrates. To eliminate the problem of long hydrolysis times, a new process engineering approach was developed where the biomass is initially hydrolyzed for 24 hours. After 24 hours, the sugars are removed and fermented separately, while the residual solids requiring further hydrolysis time are added to fresh pretreated substrate in the same tank along with fresh enzymes for further hydrolysis. This approach can significantly help reduce the biomass to sugar processing time [169].

### 5.3. Enzyme Recycling

Since biomass degrading enzymes are expensive, they need to be recycled to reduce the processing cost. Most of the cellulases have cellulose binding modules (CBMs), which anchor the enzymes to the substrate to facilitate cellulose hydrolysis. This allows unhydrolyzed solids (UHS) obtained after enzymatic hydrolysis to be efficiently used for recycling enzymes [169, 170]. However, there are certain limitations of using this method. Most accessory enzymes lack CBMs (*β*G, xylanase, xylosidase, etc.) and act on soluble substrates. Also, even enzymes with cellulose binding modules (CMBs) will eventually desorb from the substrate after a certain period of time [171]. Furthermore, enzymes can be deactivated due to thermal denaturation or shear stress. These enzyme activities will be lost after hydrolysis. When the recycled solids with bound enzymes are used for subsequent cycle of hydrolysis, they will have different enzyme profile than the one initially used. This will significantly affect the enzyme synergy during hydrolysis and will reduce the sugar conversion. Other common methods that have been used to recycle the enzymes include immobilized enzymes on nanoparticles or polymeric matrices [172, 173], ion exchange adsorption [174], and ultrafiltration [175].

### 5.4. Effect of Solid Loadings on Sugar Conversion

Enzyme conversion rate is dependent on the solid loading used during hydrolysis. It is important to note that monomeric sugars, oligomeric sugars, and degradation products produced during pretreatment also inhibit the enzymes. Most of the enzymatic hydrolysis are done to optimize pretreatment conditions [176–179] or to check enzymes activities are usually done at low solid loadings (1% glucan or 3% solid loadings). Under these conditions, the total inhibitor concentration is relatively low allowing for higher sugar conversion. As the solid loading increases, the enzyme inhibitor concentration also increases causing a drop in sugar yield (g sugar/g biomass) [122, 180]. Reduced sugar yield negatively impacts process economics. Hence, the sugar concentration and yield should be taken into account such that it is well balanced to obtain the best economics [181]. This is true for almost all pretreated substrates [182]. Also, fibers in pretreated biomass have the natural capacity to absorb water. Therefore, as the solid loading increases most of the water will be absorbed by the biomass requiring high energy to mix the biomass. Inefficient mixing creates mass transfer issues and results in lower sugar conversion. In order to avoid this problem, pretreated biomass samples are added in batches to allow enough time for the enzymes to solubilize the biomass [183]. Also, it has been reported that the power consumption during hydrolysis is influenced by the viscosity of the hydrolyzate [184].

Particle size also plays an important role during high solid loading hydrolysis [185]. Small particles result in increased surface area, which increases sugar conversion [186]. However, producing small particles requires more energy and can create issues when mixing. Small particles can agglomerate during high solid loadings, which reduces the water activity and consequently sugar conversion. A significant change in rheological properties was reported when using different sized particles during hydrolysis [187, 188]. In recent studies increased sugar conversion was evident when the particle size was increased, possibly due to reduced agglomeration and viscosity caused by the large particles [189]. These phenomena promote better mixing and allow for better sugar conversion.

### 5.5. Unproductive Oligosaccharide Production

After enzymatic hydrolysis, 15–25% of the released sugars are in the form of gluco- and xylo-oligosaccharides. As the solid loading increases, the concentration of oligomeric sugar also increases due to inhibition from high concentrations of monomeric sugar and degradation products. In the pretreatment processes that do not solubilize hemicellulose (e.g., dilute ammonia pretreatment, AFEX, etc.), more xylo-oligomers are present than gluco-oligomers. These oligomeric sugars are considered unproductive as most of the microbes used in the fermentation can consume only monomeric sugars [190]. The reason for accumulation of oligomeric sugars is unknown. Several manuscripts tend to provide explanation for such accumulation by the separation and characterization of these oligomeric sugars [191, 192]. Some possible reasons for accumulation are (i) enzyme inhibition due to degradation products and monomeric sugars, (ii) chemical modification of glucan and xylan chains during pretreatment, (iii) lack of key accessory enzymes in the enzyme cocktail that cleave specific linkages, and (iv) the presence of phenolic compounds attached to the end of the glucan chains. Furthermore, xylo-oligomers that are produced during initial stages of enzyme hydrolysis are reported to strongly inhibit biomass-degrading enzymes [193] and are considered to be responsible for oligosaccharide accumulation.

## 6. Microbial Fermentation

Microbial fermentations convert sugars produced from lignocellulosic biomass into biofuels (e.g., ethanol, butanol, acetone, isobutanol, lipids, etc.,) or biochemicals (e.g., organic acids) using fungus, yeast, or bacteria (Table 3). This process can be performed separately from enzymatic hydrolysis (separate hydrolysis and fermentation [SHF]), in combination with enzymatic hydrolysis (simultaneous saccharification and fermentation [SSF]), or combine enzyme production and enzymatic hydrolysis (consolidated bioprocessing [CBP]) [194]. Fermentation of glucose and xylose can be carried out separately or in combination (cofermentation, where both glucose and xylose are simultaneously converted). These strains do not naturally consume xylose but are made capable through genetic modification. Butanol production was previously commercialized through ABE (acetone, butanol, and ethanol) fermentation from molasses using the anearobic bacterium* Clostridium acetobutylicum* [195]. Currently, attempts are being made to produce butanol from lignocellulosic biomass either using native* C. acetobutylicum* strains or genetically modified* S. cerevisiae* or* Escherichia coli* strains [196, 197]. Some microbes like oleaginous yeasts and some algae are capable of accumulating lipids up to 75% of their body mass by consuming both glucose and xylose [198–201]. Lipids can be used to make biodiesel using transesterification process. However, lipid fermentation for fuel production based on current technology is not economical based on recently published economic analysis studies [202]. However, high-value fatty acids could potentially be a coproduct that could make biorefinery process economical [203].

### 6.1. Microbes for Producing Biofuels

**Figure 6:**
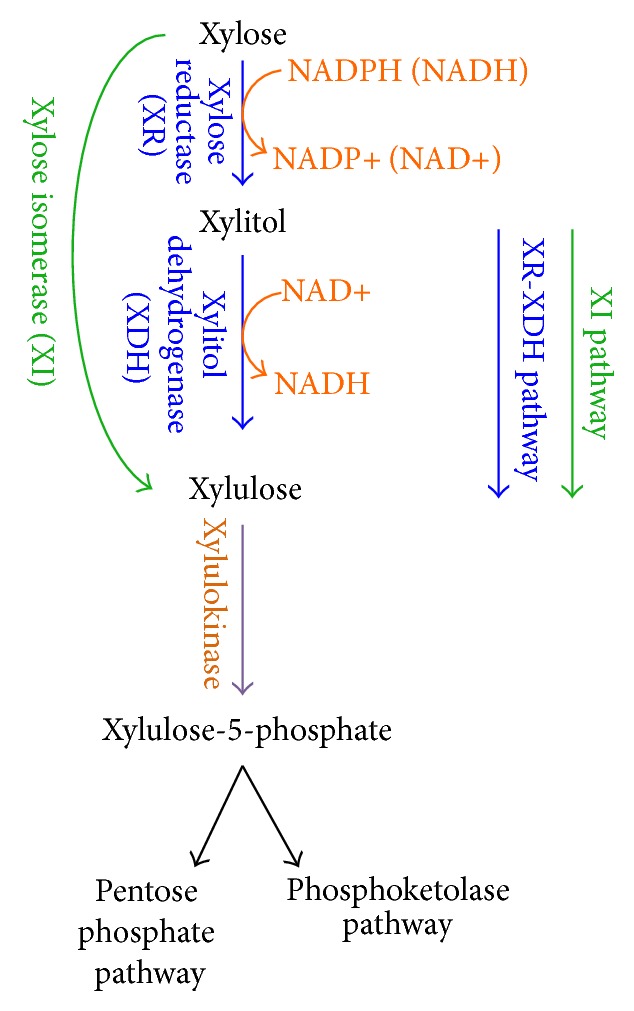
Two possible xylose metabolic pathways that are commonly used in yeast and bacteria.

Cofermentation is believed to be superior than separate fermentation in terms of cost savings. Many of challenging efforts have been made to genetically modify* Saccharomyces cerevisiae* and* Zymomonas mobilis* to enable fermentation of xylose. Two xylose-fermenting pathways have been widely studied and engineered into* S. cerevisiae* (Figure 6), namely, (i) xylose reductase (XR), xylitol dehydrogenase (XDH) pathway that exists in fungi [204, 205] and (ii) xylose isomerase (XI) pathway that exists in bacteria [206]. These two pathways convert xylose to xylulose. Xylulose is then converted to xylulose-5-phosphate which is further converted through either pentose phosphate pathway or phosphoketolase pathway [207] (Figure 6). The XR-XDH pathway, however, creates a redox imbalance under anaerobic conditions because XR prefers NADPH as the reaction cofactor, while XDH solely uses NAD^+^. Some microbes, such as* E. coli* and* Scheffersomyces stipitis* (a.k.a.* Pichia stipitis*) [208, 209], can natively ferment xylose. However, they typically have limitations, which reduce their effectiveness compared to* S. cerevisiae* or* Z. mobilis*. For instance,* E. coli* cannot tolerate high concentrations of inhibitors (degradation products and biofuels) [209] and on the other hand* S. stipiti* has a low ethanol metabolic yield [210]. Another strategy adopted by Microbiogen (http://www.microbiogen.com/) involves fermenting glucose to ethanol under anaerobic conditions and converting xylose to yeast biomass under aerobic conditions using a native* S. cerevisiae* strain MBG3248, which was screened and adapted for propagation on xylose [211]. This strategy produced 173 liters of ethanol and 134 kg of yeast protein from one ton of corn stover using dilute acid pretreatment technology.

Lignocellulosic biomass can also be directly fermented to biofuels by CBP microbes, such as* Clostridium thermocellum* [212] and* Clostridium phytofermentans*.* C. thermocellum* is a thermophilic anaerobic bacterium, which ferments at around 60°C. It produces an enzyme complex (cellulosome) to degrade cellulose, which has been shown using pure cellulose samples to be more efficient than free enzymes [213]. The fermentation products generated by* C. thermocellum* are mostly ethanol and acetic acid [213].* C. thermocellum* is a very promising CBP microbe but is limited by its low tolerance to fermentation products [214].* C. phytofermentans *is a mesophilic and an anaerobic bacterium. It produces free enzymes to degrade cellulose and hemicelluloses and can consume almost all the sugars present in the lignocellulosic biomass [215]. The main product after sugar fermentation is ethanol along with acetic acid as the minor product.* C. phytofermentans* also has a low tolerance for fermentation products [216]. CBP yeast is another focus area for researchers. The idea is to transfer novel cellulase and hemicellulase genes into a biofuel producing yeast strain (e.g.,* S. cerevisiae*). Several research works have been performed and published on this topic [213, 217–219]. White-rot fungi have also been investigated as potential CBP microbes. Recently,* Phlebia *sp. MG-60 was reported to convert 20 g/L unbleached hardwood Kraft pulp into 8.4 g/L ethanol with an ethanol yield of 0.42 g/g pulp (71.8% of the theoretical maximum) [220].

### 6.2. Challenges in Sugar Utilization (Glucose versus Xylose Uptake)

Currently, SHF is still the major process configuration for lignocellulosic biofuel production. Cofermentation microorganisms are also widely used to ferment enzymatic hydrolysate that contains both glucose and xylose. While glucose fermentation is very rapid, xylose fermentation is much slower. For instance, in a typical 6% glucan loading AFEX corn stover hydrolysate, 60 g/L glucose can be converted to ethanol by a xylose-fermenting yeast* S. cerevisiae* 424A in less than 18 hours, but the 30 g/L xylose takes an additional 96 hours to be converted to ethanol [221]. The slow xylose fermentation has been quantitatively studied by Jin et al. [209] and the reasons for such slow utilization is in part because* S. cerevisiae* does not have specialized xylose transporters so that xylose transportation relies on glucose transporters. However, glucose transporters have a high affinity to glucose and a low affinity to xylose. Therefore, glucose fermentation by* S. cerevisiae* (and most other microorganisms) always occurs prior to xylose fermentation. A considerable amount of biofuel (e.g., ethanol) present in the fermentation broth after the glucose consumption has high potential to inhibit the xylose fermentation metabolism. In addition to ethanol, other fermentation metabolites that are generated during glucose fermentation can play a critical role in inhibiting xylose fermentation. Moreover, due to the redox imbalance issue during xylose fermentation by* S. cerevisiae* 424A, the yeast cannot grow during xylose fermentation [222]. As a result, the viable cells decrease during xylose fermentation further slowing fermentation. Degradation products also reduce the specific xylose fermentation rate resulting in a reduced fermentation rate [209]. Other prominent* S. cerevisiae* strains include TMB3400 [223]; GLBRC Y35 [224]; RWB218 [225]; and DA24-16BT3 [226] (Table 3). Other* S. cerevisiae* strains produce other biofuels such as isobutanol [209, 227]; N-butanol/isobutanol [226, 228]; N-butanone/secondary butanone/isobutanol [229, 230]; and isoprenoids [230]. Some microbial strains, in some cases, consume glucose and xylose at the same time (without glucose catabolite repression) during fermentation (e.g.,* Thamnidium elegans* under aerobic conditions) [231].

### 6.3. Separation of Biofuels from Fermentation Broth

Traditionally, distillation is used to separate alcohol and water. Distillation can generate a maximum of 95% pure ethanol. Molecular sieves or additives are then needed to break the azeotrope to get pure ethanol. Although simple, distillation is an energy intensive process and requires initial ethanol concentrations greater than 4% to be economical [232]. Mostly grains or extracted sugar is used in the first generation biorefinery and therefore there are almost no degradation products in the substrate to inhibit enzymes or microbes. Hence, ethanol titer >10% are easily achievable allowing an economical distillation process. Researchers are looking at different biofuels that are insoluble in water that can be phase separated to avoid distillation process [233].

### 6.4. In Situ Biofuel Separation to Improve Fermentation Performance

**Figure 7:**
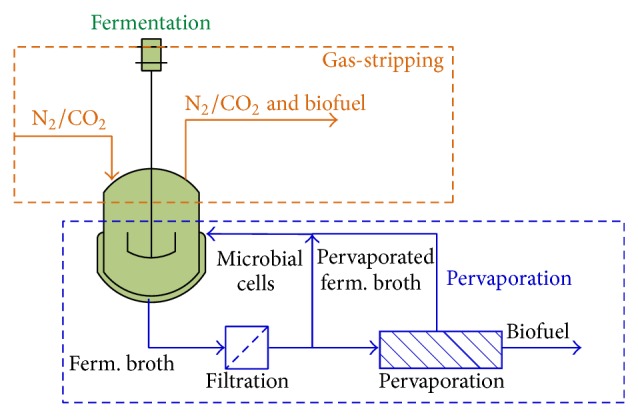
Two commonly used methods for separating ethanol from fermentation broth other than traditional distillation method in a biorefinery.

Since biofuels are known to inhibit microbes during fermentation, a large number of studies have been conducted to remove biofuels during the fermentation process. The most commonly used in situ biofuel removal methods are pervaporation and gas-striping methods. Pervaporation is a membrane-based separation technology using a nonporous or porous, hydrophilic or organophilic membrane [234]. Before the pervaporation step, there is typically a filtration step to remove microbial cells from the fermentation broth, which are then recycled back to the fermentor. The filtration step is critical because pervaporation typically operates at a temperature much higher than that tolerated by the microbial cells [235]. A fermentation broth is in contact with one side of the membrane while a vacuum or gas purge is imposed on the other side. The biofuel molecules in the fermentation broth are evaporated through the membrane (Figure 7). The pervaporated fermentation broth is then recycled back to the fermentor. Although pervaporation is a promising technology, the cost is currently higher than distillation. Vane [236] pointed out several aspects that needed to be addressed before pervaporation can become cost competitive, including energy efficiency, capital cost reduction for the pervaporation system, longer term trials using actual fermentation broths, and integration of pervaporation with fermenter. Membrane fouling by fermentation broth components is another concern when carrying out pervaporation [237].

Gas-stripping is a relatively simple process where a gas (normally nitrogen or carbon dioxide) is sparged through the fermentation broth at a high flow rate. The volatile biofuel molecules will equilibrate with the stripping gas and is passed out of the fermenter. Gas-stripping does not affect fermenting microorganisms or remove media components and is compatible with continuous fermentation processes [238]. The following are issues with gas-stripping for biofuel application: (i) large equipment cost, (ii) high energy costs to condense the stripped biofuel, (iii) increase in fermenter size, and (v) foam formation [239]. Both pervaporation and gas-stripping have been widely applied to ethanol and butanol fermentations documenting improved fermentation performance [237, 239, 240]. For instance, de Vrije et al. applied gas-stripping to remove fermentation products (isopropanol, butanol, and ethanol) during* Clostridium beijerinckii* fermentation that resulted in improved productivity [239]. Gas-stripping was also shown to greatly improve sugar utilization. For example, in a study of acetone butanol ethanol (ABE) fermentation coupled with gas-stripping, 199 g/L sugar was utilized and 69.7 g/L solvent was produced compared to sugar utilization of only 30 g/L in a control batch reactor [238].

## 7. Coproduct Generation and Its Influence of Biofuel Production Cost

Coproduct generation is very essential for producing cost competitive biofuels. For example, the first generation corn ethanol industry consists of 67% dry mill based and 33% wet mill based operations. Coproducts generated depend on the mill type [17]. Though the dry mill process produces more ethanol (2.8 gallon/bushels of corn) than the wet mill process (2.5 gallon/bushels of corn), the wet mill produces more coproducts, which results in more revenue. The corn dry mill industry produces dry distiller's grains and soluble (DDGS) and carbon dioxide as major coproducts reducing the biofuel cost by 35% [241]. In the corn wet milling process, high capital and energy intensive processing is involved (fractionating grain into starch, fiber, gluten, and germ) to produce a larger number of coproducts that include carbon dioxide, corn oil, corn gluten meal, and corn gluten feed. For the second generation technology to be able to compete with the first generation technology, several coproducts should be generated that can be sold for a high market price and subsequently reducing the overall processing costs of biofuels [242]. Some of the coproducts that could be generated from second generation biorefinery are discussed below [243].

### 7.1. Lignin

Lignin is a randomly linked aromatic polymer and constitutes about one-third of lignocellulosic biomass. Currently, the pulp and paper industry produces considerable amount of lignin that are mostly used to generate heat and power for the plant. The only product that has been successfully produced from lignin at large commercial scale is vanillin [244], which currently competes with vanillin that is produced using petrochemicals [245]. Other minor products that are produced using lignin include Bakelite (hard plastic used as utensil handles), resins and filler materials in plastic industry. With the development of several novel catalytic [246–249] or pyrolytic route [250, 251], economic uses for lignin could appear in the near future. Different pretreatments could generate different types of lignin. Removing the lignin during the pretreatment also improves downstream processing (both hydrolysis and microbial fermentation). For example, organosolv process could isolate high purity lignin [252], while other pretreatment processes like steam explosion and dilute acid pretreatment generate highly condensed lignin that could be used for producing energy products (e.g., binders for making biomass pellet, or bricket). Reliable and high quality processed lignin with adequate functionalities could be used as precursors for several products, including carbon fiber, bio-oil, resins, adhesives, polymer fillers, coating agents, plastics, paints, soil amendment, slow nitrogen release fertilizers, rubbers, elastomers, and antimicrobial agents [252–254]. However, several fractionation steps are needed to remove other components in biomass extractive (like proteins, carbohydrates) to generate pure lignin [253, 254]. If the lignin is not extracted during pretreatment, it ends up as UHS after enzymatic hydrolysis. If a portion of this could be separated into a valuable lignin stream, extra revenue could be generated for the biorefinery instead of simply burning all the UHS to provide energy for the processing steps.

### 7.2. Protein

Protein is another potential coproduct that could be separated and sold in the second generation biorefinery. Most biomass contains about 2–5% of crude protein and several early harvest grasses (e.g., switchgrass,* Miscanthus*, Napiergrass, etc.) contain 10–15% of crude protein [255]. Though several process technologies are already in place for separating the protein [256], it is economically challenging to separate crude protein from dilute water stream. Alkali as the pretreatment chemical is more favorable than acids for protein extraction [243]. Another alternative is to use proteases that cleave the proteins into amino acids. It has been estimated that when second generation biorefineries are ready to replace 10% of fossil fuels, about 100 MT/year of proteins could be produced [257]. It is widely believed that this protein could be used as a low value animal feed. There is also the potential for converting the proteins into amino acids which could be converted into organic compounds and used as precursors for synthesizing industrial products. A prominent example is the conversion of L-arginine to 1, 4-diaminobutane (precursor for Nylon 4–6) using a two-step enzymatic process [258].

### 7.3. Microbial Biomass

One of the potential coproducts in a biorefinery is microbial biomass [118]. In the batch fermentation mode, part of the microbe could be reused for the subsequent fermentation process [259]. The remaining biomass could be sold as an animal feed if using a native organism. Since most of the microbes used for fermentation in the second generation biorefineries are genetically modified organisms (that could efficiently consume both glucose and xylose) there may be some potential regulations for the use of microbial biomass as animal feed. In the semicontinuous rapid bioconversion with integrated recycle technology (RaBIT) process, the same concept may apply [169], where in the microbial cells are recycled every 24 hours for subsequent fermentation cycle. Excess cells are used for other applications. Furthermore, separated cells will contain several lignin degraded products and may lower the quality of feed [260]. Processing the proteins from these organisms followed by converting them to amino acids by acid hydrolysis is another option that could be used to produce chemicals, which can be used as precursors for making biomaterials.

## 8. Biofuels Economy of Scale and Cost

Using key process parameters developed by national renewable energy laboratory (NREL) on biomass conversions, several cost models have been developed in the past to understand the required capital investments [261–264]. With various assumptions in place, when plant capacity (0–15,000 ton per day) was plotted against total production cost ($ m^−3^), it was found that a biorefinery should handle more than 2000–5000 ton per day in order to be economical (850–875 $ m^−3^). Plants that operated at more than 5000 ton per day showed a marginal increase in the production cost [265]. However some research findings have shown that there is a steady decrease in the minimum ethanol selling price from 3.75 to 2.25 ($/gallon) as the plant size increased from 500 to 10,000 dry ton per day [266]. The cost analysis is based on several factors like biomass transportation distance, type of biomass used, type of processing technology used, efficiencies of different processing steps, type of biofuel, and coproducts production.

## 9. Water Requirement in a Biorefinery and the Necessity for Recycling

Producing biofuels using the sugar platform is a water intensive process. Water is used in almost all the processing steps. In order to meet the water requirements, important decisions should be made regarding the location of the biorefineries. In many cases, water will be pumped from the ground, which may add considerable stress to the local water resources [267, 268]. Recycling water will help to reduce this stress, but will require additional investment. Given the process and processing conditions used in the biorefineries, it will be a prerequisite to do the following when recycling the water: remove salts generated during neutralization, remove organic content, and recycle the catalysts [267]. Clarification steps have been widely adopted in the pulp and paper industry to remove suspended solids and to reduce chemical oxygen demand/biochemical oxygen demand in water. Several novel technologies have been developed to treat the water, which include biological treatment (e.g., anaerobic digestion, algal treatment), coagulation, electrocoagulation, polymer resin filtration, and coagulation-flocculation techniques. Among these techniques, coagulation (using ferric sulphate, alum, water soluble polymers, chitosan, poly aluminum chloride, fly ash, etc.) is found to be an economical approach to remove organics [269]. It is important to use minimal water in each processing step in order to reduce water recycling cost. In some cases, the choice of pretreatment in a second generation biorefinery will be determined based on the water availability in the region. Colocating the second and the first generation biorefineries will minimize waste water and reduce the stress on the water table [152, 270].

## 10. Environmental Issues

It is well reported that biofuels offers several environmental benefits over fossil fuels. Biofuels from lignocellulosic biomass have reduced emissions and fixed CO_2_, a greenhouse gas, among other things [271]. In the near future when a new biorefinery is established, several technologies will be assembled based on their impact on the environment [272]. Some of the examples are air pollution caused by particulate emission during biomass harvesting and grinding, noise pollution from explosive pretreatment processes, methods for producing pretreatment chemicals that produce GHG emissions, and release of pretreatment chemicals to the environment after processing. Life cycle analysis (LCA) is often used to assess the net environmental impact of these processing steps [273, 274]. The challenges are in carrying out accurate LCA analysis depending on the data that is collected from group of aligned processes that will be used in the biorefinery. Many companies are taking the LCA very seriously to assess the environment impact so that they could make decisions to adjust the process or areas that needed to be focused to reduce the emissions. Depending on the emissions estimated by LCA will influence the cost of establishing the biorefinery.

## 11. Energy Associated with Biomass Processing 

The goal of establishing a biorefinery is to produce energy in the form of liquid transportation fuels from biomass [275]. Therefore, it is very important to maximize the difference in energy consumed and produced. In other words, the success of a biorefinery will be evaluated by the net energy that is produced using the different processing steps [276]. Efficiencies will need to be closely monitored to maximize the net gain in the energy produced in the biorefinery [277–279]. It is widely reported in several technoeconomic evaluations of second generation biorefineries that lignin will be a good energy source for different processing steps. However, the UHS that are rich in lignin are obtained as wet slurry and will need to be dried before gasified or burned to produce energy. Hydrothermal pyrolysis is one potential technology which can use wet biomass slurry to produce bio-oil and use the heat recovered for several processing steps [280]. If lignin is extracted during pretreatment and used for producing high value products, the biorefineries will have to generate energy by burning part of the biomass that arrive at biorefineries [281]. It is widely believed that natural gas will be the primary source of energy to run biorefineries, and as the technology matures, the amount of natural gas usage will be slowly brought down. Colocating biorefineries near thermal plants (coal or nuclear plant) or using energy from renewable resources (wind, solar, geothermal, etc.) is another option to get heat and power [282]. Adding an anaerobic digestion facility adjacent to a biorefinery will be beneficial that could clean waste water and at the same time the biogas generated could be used for different biorefinery operations [283, 284].

## 12. Conclusion

This review highlighted several bottlenecks that are being faced by big corporations to commercialize the biofuel production technology (pretreatment, hydrolysis, microbial fermentation, and biofuel separation). Big corporations and oil companies that have capital to establish a new biorefinery are currently trying to align different novel process technologies that still have several separation and purification challenges to overcome [285]. The choice of pretreatment and lignocellulosic biomass could be decided based on the availability of sufficient quantity of catalyst and feedstock in that region. The aligned technologies are currently scaled up to establish pilot plants to demonstrate the feasibility. Simultaneously, biomass logistics and technoeconomic evaluations are carried out to assess the technology readiness level (TRL). Then assessments are made regarding the environment impact of using different technologies. Once appropriate feedstock, pretreatment, and enzymes are combined to produce cheap sugars, the choice of biofuels and biochemicals depends on the market demand and more importantly the biofuel policy defined by the local and federal government. Furthermore, in order to compete with the cost of petroleum fuels, the cost of biofuel processing should be kept as low as possible using energy efficient technologies and using less water. Producing as many coproducts as possible in a biorefinery will help to reduce the cost of biofuel production. If favorable conditions prevail after overcoming these hurdles, then a high capital of about 200–300 million dollars is required to establish a commercial grade biorefinery that could produce several million gallon of ethanol per year. It is important that a biorefinery should be established in an appropriate location that has good water resources, access to feedstocks, and energy that is needed to process the feedstock. Few big corporations (e.g., Abengoa, Dupont, and Poet) are putting all their resources to first establish the lignocellulosic biorefinery and then overcome all the bottlenecks to reduce the processing cost. Once this is done, then the group of technologies will be sublicensed to different biofuel manufactures. Only at this stage one can anticipate large amounts of biofuels entering the market to achieve the mandate set by DOE and USDA. To facilitate this biofuel production process, several millions of dollars are currently provided by the government to stimulate large scale biofuel production. In the past five years, only a few companies have been successful in demonstrating their technology in their pilot scales and are now gradually progressing to establish their commercial plants. Based on the public information that is available, it looks like lignocellulosic ethanol will first hit the US market, later followed by several advanced biofuels (e.g., butanol, alkanes, etc.). Due to the challenges discussed in this paper, it is anticipated that there may be a considerable delay in the commercial availability of lignocellulosic biofuels from the previously projected timeline of 2022 by the USDA and DOE.

## Figures and Tables

**Table 1:** Potential lignocellulosic biomass that is available in US and their average yield (dry ton per acre).

	Plantation in US 2012 (million acres)	Average yield (dry ton/acre)
Herbaceous agricultural residue		
Corn	90.0	3.1
Grain sorghum	7.5	1.2
Oat	3.4	1.1
Barley	4.0	2.2
Wheat	60.5	1.6
Soybean	71.5	2.3
Cotton	9.7	
Rice	3.0	4.0
Sugar cane bagasse	0.9	15.0
Dedicated energy crop		
LIHD prairie		1.8
Managed native prairie		2.5
Shawnee switchgrass		5.0
Bioenergy switchgrass		7.4
Hybrid switchgrass		9.4
*Miscanthus *		13.2
Woody residues		
Hybrid Poplar		7.0
Eucalyptus		9.0
Pine		5.1

**Table 2:** Different pretreatment technologies used in biorefinery and their advantages and disadvantages.

Pretreatment	Chemicals used	Catalyst recovered	Solid	Liquid	Process conditions	Water used	Advantages	Disadvantages	Reference
*(I) Physical *									
(1) Disk milling	NA	NA	Whole biomass	NA	Milling (10–30 mm) and grinding, particle size (0.2–2 mm)	No	(i) No chemical used, (ii) Scalable	(i) Poor sugar conversion, (ii) Highly energy intensive process	[87]
(2) Extrusion	NA	NA	Whole biomass	NA	Screw speed, 350 rpm, barrel temperature, 80°C, 40% moisture	Minimal	(i) Low temperature pretreatment and no degradation products formation, (ii) No washing and conditioning, (iii) No effluent and (iv) Can be made continuous	(i) High energy cost, (ii) More aberration of metal surface need frequent replacement	[88]
(3) Microwave	NA	NA	Enriched cellulose and hemicellulose	Glucose, Xylose	Microwave 680 W, irradiation time 24 min and substrate concentration 75 g/L	High	(i) Short processing time, (ii) High uniformity and selectivity, (iii) Less energy input than the conventional heating	(i) Cost of reactor system will be high, (ii) Additional safety requirements needed, (iii) Low sugar conversion and low substrate concentration.	[117]
(4) Acidic									
(i) Dilute sulfuric acid	Dilute sulfuric acid	No	Enriched cellulose + some hemicellulose	Soluble xylose	140–190°C, 0.4–2% sulfuric acid, 1–40 min. resident time	High	(i) Can be used for wide range of materials, (ii) Produce hydrolyzed xylose during pretreatment	(i) Need to use expensive hastealloy reactor, (ii) Difficult to controlling reaction condition, (iii) Toxic degradation products, (iv) Expensive to remove salt while recycling water	[99, 179]
(ii) Organic acid	Acetic acid or Fumaric acid or maleic acid, and so forth,	No	Enriched cellulose and some hemicellulose	Soluble lignin and hemicellulose	130–190°C, 50–90 mM of organic acid	High	(i) Fractionation of biomass in to soluble lignin rich hemicellulose stream, (ii) Low pressure reactions	(i) Recovering acid is an expensive process, (ii) High water usage to clean substrate after pretreatment	[100, 101]
(iii) Concentrated acid	Sulphurous sulfuric, HF, HCl, phosphoric acid, nitric and formic acid	Yes	Condensed lignin	Soluble glucose and xylose or Soluble cellulose which is precipitated	Shorter residence time		(i) Few cases no need enymes to depolymerize cellulose, (ii) When phosphoric acid is used cellulose I is converted to highly reactive amorphous cellulose, (iii) Effective on soft wood	(i) Corrosion, (ii) Energy intensive acid recovery step	[102]
(iv) Acidic organosolv	Methanol, ethanol, acetone, ethylene glycol and tetrahydrofurfuryl alcohol, water mixture, organic or inorganic acid	Solvent recovered, No catalyst recovery	Enriched cellulose and most of the hemicellulose	Lignin and some soluble hemicellulose	Acetone-water pretreatment (acetone : water molar ratio of 1 : 1) at 195°C, pH 2.0, 5 minutes residence time	Medium	(i) Can separate pure lignin stream, (ii) Lignin removal leads to increase cellulose digestibility	(i) High risk of high-pressure operation, (ii) Flammability, (iii) Volatility of solvents	[103]
(v) SPORL	dilute sulfuric acid, NaHSO_3 _and disc milling	Yes	Enriched cellulose and some hemicellulose	Lignin and hemicellulose	180°C, 25 min. liquor/wood = 3 : 1 v/w	High	(i) High sugar yields, (ii) Effective lignin removal, and (iii) Recovery of biomass components in less chemically transformed forms.	(i) Sugar degradation at severe conditions, (ii) Large volumes of process water used in postpretreatment washing, (iii) High costs of recovering pretreatment chemicals.	[104]
(5) Neutral									
(i) Ionic liquid	1-allyl-3-methylimidazolium-chloride ([AMIM]Cl), 1-ethyl-3-methylimidazolium-acetate ([EMIM]Ac).	99%	Enriched cellulose and hemicellulose	Lignin and some hemicellulose	100–150°C, few min. to hrs.	High	(i) Carbohydrate losses are generally low and (ii) Degradation products are significant only at severe conditions.	(i) High solvent cost, (ii) High solvent loading, (iii) Cost of solvent regeneration,	[97]
(ii) Liquid hot water	Water	NA	Enriched cellulose	Solubilized hemicellulose	160–220°C, 15 min. residence time	High	(i) No external chemical added, (ii) Simple reactor system	(i) Large water use, (ii) Some hemicellulose lost in water stream, (iii) Low solids loading	[97]
(iii) Ozonolysis	Ozone	No	Enriched cellulose and hemicellulose	Soluble lignin degraded products and some hemicellulose	Room temperature, Ozone sparging	High	(i) Effective removal of lignin and (ii) Very low production of inhibitory products, (iii) Reactions performed at atmospheric conditions	(i) Expensive due to large requirement of ozone (ii) Some portion of lignin is lost due to cleavage during ozone pretreatment process	[98]
(6) Alkaline									
(i) AFEX	Liquid or gaseous anhydrous ammonia	Up to 97%	Whole biomass	NA	100–140°C, 1 : 1–2 : 1 ammonia to biomass loading, 30–60 min. residence time, 60–100% moisture.	Medium	(i) Volatile ammonia can be recovered and reused, (ii) Lesser degradation product formation, (iii) Dry to dry process, (iv) Lignin relocation to surface help to densify the biomass	(i) Safety precautions for handling ammonia, (ii) Ammonia recovery step is added cost, (iii) Not efficient for hardwood biomass.	[89, 126, 132]
(ii) ARP	Ammonium hydroxide	No	Enriched cellulose and some hemicellulose	Soluble lignin and hemicellulose	160–180°C, 10–30 min. residence time, 0.5 g ammonium hydroxide per g of biomass	High	(i) Recalcitrant lignin can be removed, (ii) Works very well for grasses	(i) High amount of water used in the process, (ii) Energy intensive process, (iii) Not efficient for hardwood biomass.	[90]
(iii) SAA	15% ammonia solution	No	Enriched cellulose and some hemicellulose	Soluble lignin and hemicellulose	Solid to liquid ratio 1 : 11, 60°C, 8–24 h,	High	(i) Lower reaction temperature	(i) Longer Residence time, (ii) Large water usage, (iii) Scale-up issues.	[91]
(iv) NaOH	NaOH	NaOH recovered	Cellulose II formation and some hemicellulose	Soluble lignin and hemicellulose		High	(i) Conversion of highly reactive cellulose II (ii) Solubilization of lignin	(i) Longer residence time, (ii) Large water usage, (iii) Scale-up issues, (iv) Expensive catalyst recovery	[92]
(v) Alkaline H_2_O_2_	NaOH, H_2_O_2_	NaOH recovered	Enriched Cellulose and some hemicellulose	Soluble degraded lignin and hemicellulose	0.5–2% sodium hydroxide, 0.125 g H_2_O_2_/g biomass, 22°C, and atmospheric pressure for 48 h.	High	(i) Milder pretreatment condition. (ii) Commercially used in paper industry and scalable,	(i) Large water use, (ii) Catalyst recovery is expensive, (iii) Energy content of lignin is lost due to oxidation.	[93]
(vi) Lime	CaO with and without oxygen	No	Whole Biomass	NA	25–160°C, 120 min. to weeks, 0.07–0.2 g CaO/g biomass	High	Pretreatment can be done using inexpensive pretreatment reactor system	(i) Large water use, (ii) Catalyst recovery is expensive, (iii) Residence time is longer	[94]
(vii) Alkaline wet oxidation	Oxygen or air	No	Whole Biomass	NA	>120°C, 0.5–2 Mpa, <30 min. residence time.	Less	(i) Dry to dry process (ii) Lesser degradation products formation	(i) Require high pressure equipment, (ii) High cost of oxygen that is used as a catalyst, (iii) Oxidation of lignin makes is lesser dense in energy	[95]
*(II) Physiochemical *									
(1) Steam explosion (catalyzed using SO_2_)	Steam and SO_2_	No	Enriched cellulose	Soluble hemicellulose	180–210°C, 1–120 min. residence time	High	Works well both for hardwood and herbaceous biomass	Expensive reactor system requirement due to high pressure operation	[105]
(2) Supercritical CO_2_	CO_2_ (21.37 Mpa) + Water = Carbonic acid	NA	Whole biomass	No	112–165°C, 0–73% moisture, 10–60 min. residence time	Medium	(i) Less corrosiveness, (ii) Nontoxic chemical, (iii) Non-flammability, (iv) No waste stream	(i) High pressure reactions, (ii) Need expensive reactor system.	[106, 107]
*(III) Biological *	Microbes like fungus or bacteria	No	Whole biomass (with reduce cellulose and hemicellulose content)	NA	25–30°C, solid state fermentation, 80–120% moisture, 10–15 days residence time	High	(i) Mild pretreatment condition, (ii) Low energy consumption, (iii) No chemicals needed	(i) Slow process and slow throughput, (ii) Sugar conversion after pretreatment is not high, (iii) Larger space requirement and (iv) Need continuous monitoring	[108, 109]

Abbreviations: rpm: revolutions per minute; W: watt; NA: not applicable; L: liter; g: gram; C: centigrade; mm: millimeter; min: minutes; mM: millimolar; h/hrs: hour/hours; V/W: volume/weight; Mpa: mega Pascal, ha: hectare.

**Table 3:** Examples of microbial strains that are used for biofuel production.

Strain name	Strain description	Biofuel type	Titer (g/L)	Yield (g/g consumed sugar)	Reference
*S. cerevisiae* 424A (LNH-ST)	Xylose and glucose fermenting strain. Incorporated XR and XDH genes from * S. stipitis* and over-expressed endogenous xylulokinase gene	Ethanol	45	0.4	[222]

*S. cerevisiae* TMB3400	Same as above	Ethanol	33	0.51	[223]

*S. cerevisiae* GLBRC Y35	Same as above	Ethanol	46	0.49	[224]

*S. cerevisiae* RWB 218	Xylose and glucose fermenting strain. Incorporated XI gene from piromyces; overexpression of endogenous xylulokinase, ribose 5-phosphate isomerase, ribulose 5-phosphate epimerase, transketolase and transaldolase genes; knockout of GRE3 gene, which encodes an aldose reductase.	Ethanol	47	0.38	[225]

*S. cerevisiae *DA24-16BT3	Xylose, glucose and cellobiose-fermenting strain. Incorporation of XR and XDH genes for xylose fermentation, and cellodextrin transporter and intracellular *β*-glucosidase genes for cellobiose consumption	Ethanol	60	0.38	[205]

*E. coli* KO11	Homoethanolic fermentation strain. Incorporated pyruvate decarboxylase and alcohol dehydrogenase genes (PET operon) from *Zymomonas mobilis*.	Ethanol	40+	0.44~0.51	[208]

*Zymomonas mobilis *AX101	Genetically engineered xylose, arabinose and glucose fermenting strain	Ethanol	42+	0.42 (estimated)	[196]

*S. stipitis* FPL-061	A mutant selected for growth on L-xylose in the presence of respiratory inhibitors	Ethanol	29	0.42	[286]

*Clostridium thermocellum* LQRI	Native CBP strain	Ethanol	1.4	0.26	[212]

*Clostridium phytofermentans* ATCC 700394	Native CBP strain	Ethanol	2.8	0.39	[216]

*Clostridium acetobutylicum* P262	Native acetone, butanol and ethanol producing strain	Acetone, butanol, and ethanol	2.9/8.1/0.3	0.39	[195]

*Clostridium beijerinckii* NRRL B593	Native isopropanol, butanol and ethanol producing strain	Isopropanol, butanol, and ethanol	3.2/6.9/0.45	0.32	[241]

*E. coli strains *	Abiotic long chain keto acids and alcohols producing strains, achieved through extending branched-chain amino acid pathways	1-Propanol, isobutanol, 1-butanol, 1-pentanol, and so forth	0.007~1.2	N/A	[197]

*Saccharomyces cerevisiae *	Overexpression of genes in valine metabolism	Isobutanol	<0.1	0.00097	[227]

*Saccharomyces cerevisiae *	Gevo strains for n-butanol and Isobutanol production; incorporated butanol synthetic pathway from Clostridia species; Built isobutanol pathway either in mitochondria or in the cytosol using endogenous or heterologous genes	n-butanol/isobutanol	N/A	N/A	[226, 228]

*Saccharomyces cerevisiae *	Butamax/Dupont strains for n-butanol, sec-butanol, and isobutanol production. Incorporated many different heterologous genes and endogenous genes to build butanol synthesis pathways in either mitochondria or cytosol.	n-butanol/sec-butanol/isobutanol	N/A	N/A	[229, 232]

*Saccharomyces cerevisiae *	Amyris strain for isoprenoid production. Strong expression of Mevalonic acid (MVA) pathway genes and manipulation of many other genes	isoprenoid	N/A	N/A	[232]

*Rhodosporidium Toruloides* Y4	Lignocellulosic hydrolysate domesticated strain that could consume both glucose and xylose	Lipids	5.5	0.10	[198]

*Cryptococcus curvatus *	Native oleaginous yeast that could consume both glucose and xylose	Lipids	5.8	0.20	[199]

*Lipomyces starkeyi *	Native oleaginous yeast that could consume both glucose and xylose	Lipids	4.6	0.16	[199]
